# Anticipating the Impact of AI on Diet and Exercise Apps: Foresight Study Applying the Futures Wheel

**DOI:** 10.2196/96285

**Published:** 2026-07-16

**Authors:** Xavier Tadeo, Gyula Seres, Sapphire Lin, Yoann Sapanel, Han Shi Jocelyn Chew, Dean Ho

**Affiliations:** 1 Institute for Digital Medicine (WisDM), Yong Loo Lin School of Medicine, National University of Singapore, 28 Medical Drive, #05-COR, Centre for Life Sciences, Singapore, 117456, Singapore, 1 6601 7766; 2 The N.1 Institute for Health (N.1), National University of Singapore, Singapore, Singapore; 3 Singapore’s Health District at Queenstown, Yong Loo Lin School of Medicine, National University of Singapore, Singapore, Singapore; 4 Department of Biomedical Informatics, Yong Loo Lin School of Medicine, National University of Singapore, Singapore, Singapore; 5 Alice Lee Centre for Nursing Studies, Yong Loo Lin School of Medicine, National University of Singapore, Singapore, Singapore; 6 Behavioural and Implementation Science Interventions, Yong Loo Lin School of Medicine, National University of Singapore, Singapore, Singapore; 7 Department of Biomedical Engineering, College of Design and Engineering, National University of Singapore, Singapore, Singapore; 8 Department of Pharmacology, Yong Loo Lin School of Medicine, National University of Singapore, Singapore, Singapore; 9 The Bia-Echo Asia Centre for Reproductive Longevity and Equality, Yong Loo Lin School of Medicine, National University of Singapore, Singapore, Singapore

**Keywords:** mobile apps, health promotion, fitness centers, healthy aging, behavioral sciences, forecasting, consumer health information, biomedical technology assessment, Futures Wheel, longevity, AI

## Abstract

AI has entered the wellness space through apps and wearables. These technologies can collect real-time data, infer lifestyle patterns, and dynamically generate nutrition and exercise recommendations. Generative AI personalizes diet and activity information, encouraging behavior change. The objective of this Viewpoint is to explore the potential medium-term consequences of AI integration in diet and exercise apps from an end-user perspective. We applied a foresight methodology—the Futures Wheel (FW)—and defined its central trend as the growing integration of AI into consumer wellness platforms. The analysis outlines seven first-order consequences: (1) personalization of nutrition and fitness plans, (2) 24/7 health coaching, (3) integration with smart technology, (4) increased privacy and surveillance concerns, (5) data-driven risk profiling and moral hazard, (6) incorporation into organizational processes, and (7) acceleration of health inequalities driven by the digital divide. Second-order consequences included potential improvements in health outcomes and health literacy, as well as risks of privacy erosion, algorithmic bias, behavior-linked underwriting models, deskilling of health and fitness professionals, and shifts in food and exercise culture toward more individualized, and potentially isolating practices. Cross-cutting patterns highlighted recurring trade-offs between personalization and surveillance, scalability and user agency, and optimization and equity. Wellness practice will expand along with AI’s ability to personalize recommendations, automate behaviors, and engage users. AI wellness popularization is promising for chronic disease prevention and health optimization. The FW reveals that the depth of adaptation will be determined by the implementation of changes at the levels of technology, user behavior, infrastructure, and legal and ethical frameworks.

## Introduction

AI is increasingly used in consumer wellness technologies for health-related purposes [1]. Digital platforms can collect continuous behavioral and physiological data, infer lifestyle patterns, and deliver personalized recommendations [2]. Advances in hardware and generative AI enable real-time feedback loops that can adapt nutrition and physical activity guidance to individual goals, preferences, and contexts. As a result, AI-powered wellness apps are becoming persistent, interactive agents in users’ daily lives rather than passive self-monitoring tools. The widespread ownership of smart devices enables a rapid expansion of AI-enabled diet and exercise apps. A recent study estimates that more than 600 million users own wearables capable of tracking their heart rate and step counts, and 300 to 500 million users own a sleep-tracking device [3]. Consumer demand creates opportunities for scalable behavior change support for weight management, physical activity promotion, and chronic disease prevention [4].

As consumer wellness technologies increasingly influence prevention, behavior change, and chronic disease risk management, they represent an important frontier for medical futures research. These technologies are often framed as a form of preventive health intervention that may reduce reliance on clinical care and mitigate growing health care costs. At the same time, their widespread adoption introduces complex challenges related to data privacy, algorithmic bias, user agency, and unequal access to digital resources [5,6]. As AI systems increasingly shape everyday health behaviors outside traditional medical settings, understanding their broader and longer-term consequences for users has become an urgent research priority. This demand aligns with public health priorities supporting healthy longevity in an aging society.

In the context of this rapidly changing landscape with well-identified actors and emerging technologies, there is a need for a structured overview of possible consequences and implications. Futures studies, an interdisciplinary discipline that uses different foresight methodologies to explore and anticipate future developments, provide a forward-looking framework for exploring the future of diet and fitness apps powered by AI [7]. Futures methodologies have been used for decades in many fields, but they remain underutilized in the health care domain [8]. Given the accelerated technological progress in health care and the subversion of health care roles brought about by digital health, a systematic study of the future of wellness apps is warranted. This paper aims to investigate the future of AI in diet and exercise apps using a foresight methodology known as the Futures Wheel (FW)—a strategic foresight tool that visually maps the direct and indirect consequences of a central trend—by identifying first- and second-order ripple effects that form the consequences of AI integration into consumer health apps [9,10]. The FW is particularly suited to the analysis of a user-facing digital health technology domain where complex interactions generate nonlinear effects and several potential scenarios.

## Foresight Methodology

### Study Design

In this study, we used a qualitative foresight methodology—the FW—to explore the potential medium- to long-term effects of AI integration in consumer wellness apps, specifically diet and exercise apps. The FW is a structured, participatory technique designed to identify and organize the direct and indirect consequences of a defined central trend by mapping successive layers of impacts [11]. It is an established participatory foresight method in which a central trend is defined, and participants create a list of their perceived first- and higher-order consequences expected to emerge from that trend.

We focused the analysis on end users of the apps rather than other actors such as developers, regulators, and the overall health care system. First-order consequences of the trend stem from the central phenomenon and are linked to it with spokes; together they are visualized as a wheel. Second-order consequences were defined as downstream effects arising from each first-order consequence and were represented as a subsequent ring. We explore potential effects within a 5-year horizon.

The FW was developed through structured consequence mapping and iterative synthesis conducted by the study’s authors (XT, GS, and SL) as panel members. First-order consequences were generated independently and subsequently discussed in group sessions, during which overlapping or redundant impacts were merged or discarded. All members reached consensus on which consequences to include and how to classify them. The subsequent selection of second-order consequences iteratively followed the same procedure. We redefined the wheel over several iterations to improve its coherence and internal consistency.

To connect the wheel with the existing knowledge base, we expanded the consequences with the prevailing scientific literature, policy reports, and publicly disclosed information on product development efforts. This step helped us contextualize the impacts without attempting a comprehensive review of all available empirical evidence. The final FW served as the analytical framework for organizing and interpreting the main findings.

### Panel Composition

The FW was developed by a 3-member expert panel consisting of the study’s authors. The panel was intentionally interdisciplinary to capture technical, behavioral, and socioinstitutional dimensions of AI integration into wellness apps. The panel included a biologist with expertise in metabolic health and digital biomarkers; a behavioral economist with expertise in decision science, incentives, and health behavior change; and a communication scientist specializing in digital media ecosystems and user-technology interaction. The panel size (n=3) aligns with the exploratory nature of the FW method, which is designed for structured consequence mapping. Members were selected to provide complementary expertise across biological, behavioral, and technical dimensions of AI-enabled wellness systems. To mitigate potential bias, the implemented safeguards included independent generation of first-order consequences, iterative group deliberation, and unanimous inclusion criteria. All panel members had prior research experience related to digital health technologies. Given the exploratory and methodological focus of the study, these 3 authors themselves served as the elicitation panel.

### Elicitation Procedure

The FW used a structured, multistage elicitation process conducted over 3 iterative working sessions (each approximately 60‐90 min) across a 6-week period.

#### Stage 1: Central Trend Definition

The panel first defined and operationalized the central trend: increasing integration of AI into consumer wellness apps for personalized nutrition and fitness guidance. The scope was limited to consumer-facing digital platforms (eg, mobile apps integrated with wearables) but excluded clinical decision support systems and AI integrated into the clinical workflow (eg, diagnostics).

#### Stage 2: Independent First-Order Consequence Mapping

Panel members independently generated potential first-order consequences, defined as direct and immediate outcomes resulting from widespread AI-driven personalization. Independent generation was used to reduce anchoring effects and groupthink. Ideas were submitted in writing prior to group discussion. The panel members used generative AI (ChatGPT [OpenAI] and Gemini [Google Inc]) for individual brainstorming at this stage only. AI-generated ideas were critically evaluated, reformulated, and either discarded or validated by the respective member before submitting them to the rest of the panel. These tools were used to widen divergent thinking. Inclusion required consensus independent of AI origin. AI was not used in classification, validation, or final structuring.

#### Stage 3: Group Deliberation and Expansion

During structured workshops, independently generated consequences were pooled, clarified, and discussed. Duplicates were merged, ambiguities resolved, and conceptual overlaps identified. For each retained first-order consequence, the panel then generated second-order consequences—defined as indirect, systemic, or emergent effects resulting from the first-order impacts.

The group followed a forward expansion logic: each consequence was examined using the prompt, “If this becomes widespread and normalized, what structural or behavioral changes follow?” We continued this process until a point of theoretical saturation, where no substantially new ideas emerged. Disagreements were resolved through structured discussion. Items that lacked a clear causal justification were either reformulated or removed. No voting system was used; inclusion required unanimous agreement that was reached for all items appearing in the manuscript.

### Consolidation and Thematic Structuring

Following expansion, second-order consequences were consolidated through thematic coding. Items were grouped into broader domains (eg, technology, user behavior, infrastructure, and the legal and ethical framework). Consolidation decisions required consensus among all 3 panel members. Disagreements were resolved through discussion. If consensus could not be reached immediately, the item was provisionally retained and revisited in a subsequent session. Final inclusion required unanimous agreement.

To enhance coherence and prevent redundancy, consequences were refined to ensure conceptual distinctiveness, logical traceability from the central trend, and noncircular causal structure. This consolidation process resulted in a structured 2-tier FW comprising first- and second-order consequences.

The evolution from initial expert-generated concepts to consolidated first-order effects is summarized in Table S1 in Multimedia Appendix 1; some concepts were reclassified as higher-order effects during this process.

### Analysis

Following the construction of the FW, an evaluative analysis phase was conducted. Collectively, the participants reviewed all consequences and assessed them for internal consistency and relevance. In this phase, consequences were iteratively revised, allowing impacts to be merged, reframed, reclassified across orders, or removed when deemed redundant or insufficiently linked to the central trend. At a higher level, the analysis also aimed to identify recurring patterns, reinforcing dynamics, and trade-offs across different branches of the wheel. We identified patterns by comparing clusters of consequences, rather than applying any quantitative weighting or prioritization.

Wheel editing followed the Wagschal [12] rule of unanimity, whereby impacts are included by consensus [13]. This rule ensures that the wheel’s effects are reasonable and that conclusions are not too speculative and have limited predictive value. Each effect was analyzed and supported with related references from scientific journals, gray literature, and informal sources that capture emerging signals or weak trends (refer to Multimedia Appendix 1 for expanded methodology and contextualization of primary and secondary effects). The finalized FW and the identified patterns informed the organization and narrative structure of the upcoming Results section. First-order consequences are presented as primary categories, with associated second-order consequences discussed within each category to illustrate downstream processes.

## Anticipated Consequences of AI Integration

### Overview

To explore the potential trajectories of AI integration in diet and exercise apps, a central trend was defined: the growing integration of AI in consumer wellness platforms.

The analysis yielded seven first-order consequences: (1) personalization of nutrition and fitness plans, (2) integration with smart technology, (3) increased privacy and surveillance concerns, (4) data-driven risk profiling and moral hazard, (5) acceleration of health inequalities driven by the digital divide, (6) incorporation into organizational processes, and (7) 24/7 health coaching. Second-order consequences are listed and discussed under each first-order effect.

The identified first-order consequences follow a magnifying pattern. Their order illustrates the overall scope of the subject: keeping the focus on the user perspective, the list starts with the individual impact, continues with the related stakeholder interactions, and concludes with its positioning in society.

The second-order consequences were defined following 2 key principles. The first principle is purely methodological: each second-order consequence is linked to a first-order consequence. The second principle is that each second-order consequence falls into 1 of 4 key domains relevant to the impact of new technology and its adaptation. These are (1) technological, (2) infrastructural, (3) behavioral, and (4) ethical consequences. The numbering follows a chronological order. Technological development is a precursor to all other domains. The infrastructural domain is necessary for implementation. The penultimate step is how users are expected to behave. Finally, ethics govern the societal aspects of new technologies.

The selection of the final list of second-order consequences followed this domain-specific categorization. For every first-order consequence, the panel considered whether lower-level consequences exist in each of the domains. The final step was establishing whether causal links exist between the second-order consequences.

**Figure 1.**
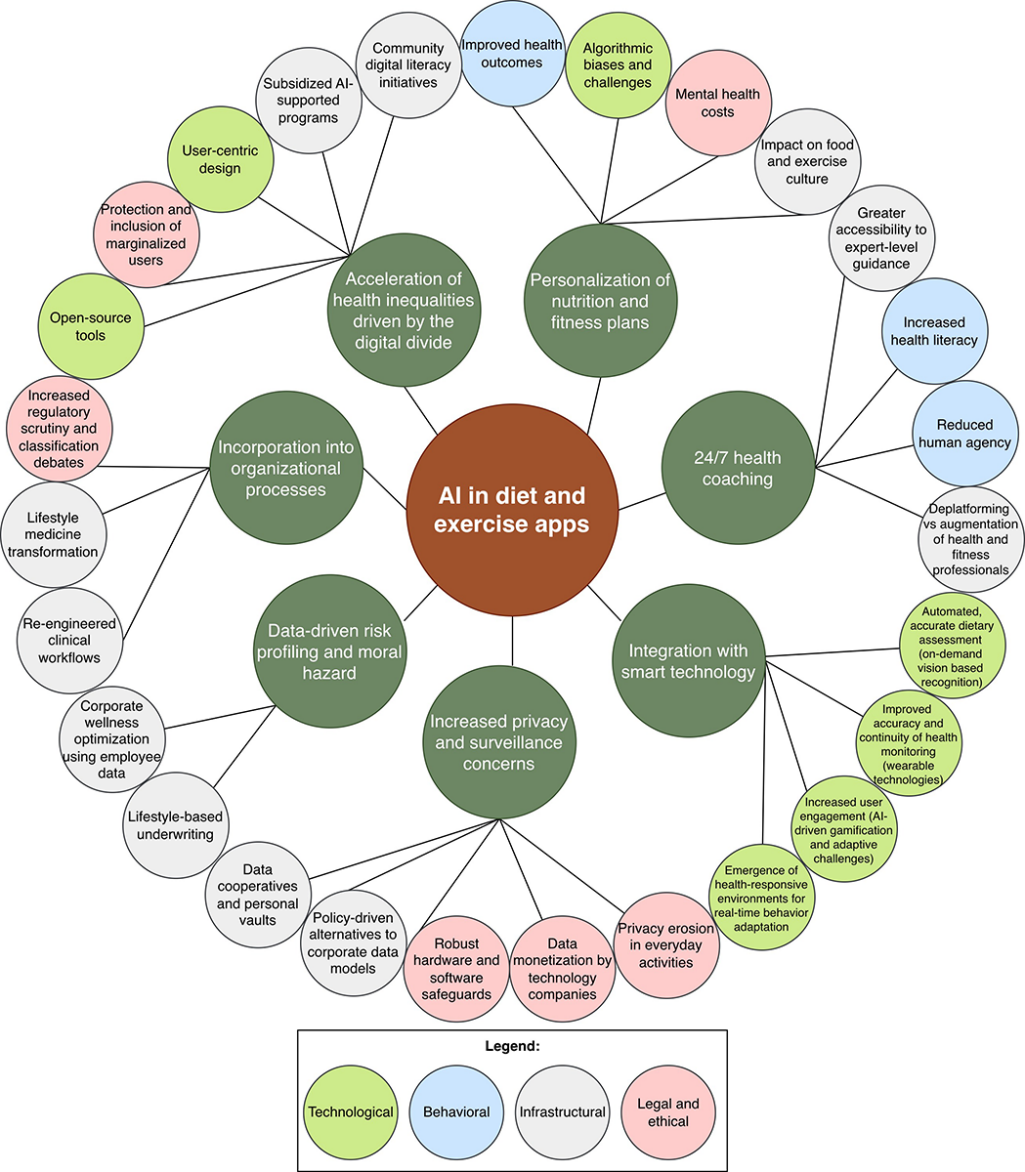
Futures Wheel of potential impacts of AI in diet and exercise apps. The central trend is depicted, connected to 7 nodes that represent first-order consequences. Downstream, second-order consequences arise from each first-order consequence. Second-order consequence colors indicate their dominant dimension—either technological, behavioral, infrastructural, or legal and ethical.

The resulting FW is depicted in Figure 1. The finalized FW maps the medium-term consequences of increasing AI integration into consumer diet and exercise apps from an end user perspective. The central node of the wheel is defined as the growing integration of AI into consumer wellness platforms, encompassing AI-driven tracking, coaching, and predictive analytics. The wheel is structured in 2 concentric layers. First-order consequences radiate directly from the central trend and represent immediate, domain-level impacts experienced by users. These first-order effects are connected to the central trend through spokes and collectively form the inner ring of the wheel. Second-order consequences form the outer ring and represent downstream effects arising from each first-order consequence. Each second-order consequence is explicitly linked to a corresponding first-order category, preserving causal traceability.

### Domain Analysis

As illustrated in Figure 1, the second-order consequences are grouped according to domains defined by the stakeholder level. The ordering corresponds to the distance from technology. Because these technologies directly interact with users, the second domain is user behavior. The next domain is the broader implementation pathway through infrastructural developments, and the final domain is the broader societal impact in the form of the legal and ethical framework. Considering these domains reveals an interesting pattern that not only highlights the impact of new technologies but also illustrates why the FW is an appropriate analytic tool for user-facing technologies. Table 1 shows 3 implementation levels of AI-enabled diet and fitness apps across the 4 domains, with the possible overall impact in each in a 5-year timeframe.

In all domains of the second-order consequences, it is plausible to observe different levels of implementation within our time frame. In the limited implementation scenario, the domain will not substantially change from the status quo. The more advanced intermediate implementation scenario comes with further expansion of consequences in scope and depth. The most radical deep implementation scenario covers massive new developments and integration of the new technologies in the respective domain.

The main finding of this framework is that these scenarios do not necessarily overlap and may develop asynchronously. It is possible, for example, that the legal framework reaches the deep implementation stage before or without technology or user behavior reaching the same level of implementation. Such a gap can be created with proactive governance and regulatory efforts. This sort of independence precludes a conventional vision writing or scenario analysis that encompasses a small number of complete descriptions of near-future developments [14]. Importantly, each implementation level carries not only expanded capabilities but also distinct failure modes and governance challenges—the table reflects both the potential of each stage and the conditions under which that potential may be undermined.

**Table 1.** Three implementation levels of AI-enabled diet and fitness apps across 4 domains: technology, user behavior, infrastructure, and legal and ethical frameworks.

Domain	Limited implementation	Intermediate implementation	Deep implementation
Technology	AI-enabled diet and fitness apps integrate basic personalization, wearable tracking, and conversational coaching Personalization relies predominantly on self-reported data Systematic inaccuracies and biases invisible to users but consequential for recommendation quality	Multimodal sensing (wearables, smart glasses, continuous glucose monitors, and Internet of Things devices) Systems predict health states and behavioral triggers Interoperability challenges emerge as health IT infrastructures lack standardized protocols to unify inputs across devices and platforms	Highly integrated AI wellness ecosystems capable of anticipatory interventions and continuous optimization of diet, activity, sleep, and mental health The boundary between wellness prediction and medical diagnosis requires explicit regulatory definition Clinical validity thresholds and accountability remain unresolved
User behavior	Users adopt AI-driven coaching as a supplement to existing health practices Improved health literacy and motivation through feedback loops Early adoption skews toward already health-motivated and digitally literate users Initial effect may reinforce rather than reduce existing health engagement gaps	Behavioral routines guided by algorithmic recommendations Reliance on AI-mediated goal setting and nudging grows Habitual algorithmic nudging risks behavioral lock-in, and the capacity for autonomous self-regulation progressively eroded beyond simple app dependency	Health decision-making partially externalized to AI systems, potentially affecting autonomy, intrinsic motivation, and social health practices System failure, withdrawal, or inaccessibility carries disproportionate risk for populations that have deskilled self-health management
Infrastructure	Stand-alone apps and wearable ecosystems dominate the market Data flows remain largely platform-specific The absence of data portability standards creates platform lock-in Users switching platforms lose behavioral history, favoring incumbents over user interests	Integration across digital health ecosystems, including insurance programs, workplace wellness systems, and preventive health care initiatives Cross-sector integration contingent on harmonization of data formats and consent frameworks across health, insurance, and workplace systems, which remain unresolved in most markets	AI wellness systems embedded in institutional infrastructures (public health, insurance underwriting, corporate wellness, and clinical prevention programs) Deep institutional embedding introduces systemic resilience risks Platform discontinuation, cyberattacks, or infrastructure failures carry population-scale consequences
Legal and ethical framework	Regulatory attention to privacy, transparency, and algorithmic accountability in consumer health technologies Active instruments at this stage include the European Union AI Act’s high-risk classification provisions, the World Health Organization AI health governance principles, and emerging US state-level algorithmic accountability bills, which collectively define the current regulatory floor	Regulatory frameworks address data governance, algorithmic bias, and fairness in AI-driven health services Liability allocation remains unresolved No existing framework clearly designates responsibility when an AI wellness recommendation contributes to harm	Codified legal frameworks regulate AI-mediated health decision-making, protect user autonomy, and manage societal impacts such as inequality and surveillance Legal framework may reach deep implementation asynchronously with technology Both proactive governance and regulatory lag carry distinct risks for user protection

## Discussion

The FW proved well suited to the analysis of consumer-facing AI wellness technologies: its consequence-mapping structure accommodated the nonlinear and multistakeholder nature of AI wellness technology diffusion, while the 2-tier causal architecture maintained traceability between the central trend and downstream effects. The FW is well positioned to reveal structural interdependencies across consequences rather than converging on consensus predictions—an advantage in an area characterized by high uncertainty and rapidly shifting technological baselines.

Our FW shows that integration of AI into wellness apps may generate cascading effects across behavioral, technological, institutional, and ethical domains. These effects do not point to a univocal future but to multiple interacting trajectories, including improvements in health outcomes, surveillance risks, institutional adaptation, and digital inequality.

At a user level, the most immediate first-order consequences identified by the wheel are the individualization of nutrition and fitness plans and constant health coaching, made possible by AI’s increasing data analysis and adaptive recommendation capabilities. For the same reason, improvements in healthy behavior adherence and preventive health scalability are expectable. Passive tracking tools become agents that identify behavioral triggers or early signs of health issues. Second-order consequences, however, warn of the psychological risks of AI wellness implementation. Technostress, dependency, and reduced intrinsic motivation may be the trade-offs of an otherwise health-promoting platform.

The wheel highlights technological convergence as another enabler of future developments. The integration of wearables, smart environments, and AI analytics expands wellness into new environments with ambient intelligence.

Demands for data protection are likely to increase, as AI platforms continuously collect sensitive data. Monetization, surveillance, or user profiling may be another trade-off of AI expansion into wellness. In parallel, protective technical and legal frameworks will be developed, but the tension will persist. In the same vein, the FW identified challenges in data-driven risk profiling and institutional use of behavioral data. Insurance underwriting and corporate wellness programs may incentivize healthy behaviors, but there is a risk of discrimination against those with socioeconomic constraints or disabilities, who might face higher premiums or workplace penalties. As illustrated in the domain analysis, the risks identified in this branch of the wheel are unlikely to be meaningfully constrained until the regulatory and ethical domain reaches at least an intermediate implementation level—suggesting that proactive regulatory action, rather than technological self-correction, is the more plausible near-term safeguard.

AI platforms meant to promote health may intensify health inequality. Mobile device ownership, platform subscription, and digital skills may contribute to the digital divide within the population. Equalizing initiatives also emerge on the wheel, such as subsidized devices or inclusive design.

AI wellness technologies may change practices related to food and exercise. Individual diets that ignore social context complicate communal eating, and individual routines may clash with group activities. Health optimization may take precedence over social enjoyment, altering cultural perceptions of eating or exercising.

The FW suggests that the expansion of AI wellness may be shaped by institutional and regulatory responses. The frontier between consumer tools and digital therapeutics may become increasingly blurred, prompting institutions to build frameworks for safety, transparency, and accountability. Institutional integration brings scale, sustainability, and trust, and will play a role in legitimizing AI presence in population health strategies. Conversely, institutional interests may conflict with particular ones, raising concerns about matters such as consent, privacy, and surveillance.

AI demand for computational power is increasing, prompting concerns around carbon dioxide emissions, water use, and rare mineral extraction. However, quantifications vary [15-18]. To some extent, these developments could diminish the positive implications of AI wellness. Because of its uncertain and cross-cutting nature, this effect was not included in any FW branch.

Users are the focus of this study, but other stakeholders will also play an important role in shaping and supporting the implementation of AI wellness solutions. The consequences outlined by the FW will require that developers increasingly include users in the design phase, mitigate algorithmic bias, and preempt negative behavioral effects. Regulators will need to create adaptive frameworks that do not stifle creativity but address safety and indication creep. Health care providers will have to appraise the integration of AI apps into clinical workflows, especially regarding logistics and data reliability.

In the future, improvements will necessarily happen in algorithm accuracy for diverse users. To enhance user experience and weight loss outcomes, AI chatbots will probably be designed to be human-like and context-aware. Apps will integrate health data, user traits, behavioral patterns, and emotional states to deliver effective, tailored recommendations. As predictive models grow more precise, apps may begin delivering real-time interventions based on subtle behavioral cues or environmental data. On the other hand, AI is expected to become more adaptive and persuasive and might blur the line between support and manipulation. User agency and informed consent might inform the future ethical design of feedback systems. International policy alignment through the Organisation for Economic Co-operation and Development (OECD) AI frameworks or the European Union Health Data Space may materialize in equitable and safe AI development.

Taken together, the first- and second-order effects of this FW indicate that we might be approaching a sociotechnical transformation rather than a technological shift. Apart from users, the consequences affect institutional structures, data governance, and cultural norms. A thoughtful implementation should maximize the potential of AI integration in lifestyle routines while ensuring ethical and sustainable use.

## Methodological Strengths and Limitations

Rigor in the foresight process was aided by multiple safeguards. First, the panel members independently generated first-order consequences before the initial group discussion to reduce potential anchoring and promote diverse viewpoints. Second, the FW followed a predefined 2-tier causal structure linking first-order and second-order consequences to the central trend, ensuring traceability. Third, consequences were included only after unanimous agreement. This principle provides a plausibility filter intended to exclude effects considered insufficiently justified or speculative. The interdisciplinary composition of the panel enabled consideration of physiological, behavioral, and sociotechnical dimensions of AI-enabled wellness technologies.

Nevertheless, certain limitations should be acknowledged. First, the panel consisted of 3 experts who were also the study authors, which may limit the diversity of perspectives and introduce interpretive bias. Second, the FW method identifies plausible chains of consequences rather than estimating their likelihood or magnitude. Thus, the results should be interpreted as structured foresight propositions rather than predictive claims. Third, the analysis also focused exclusively on end user perspectives. This limited scope leaves out potential consequences affecting other stakeholders such as developers, regulators, or health care providers. Given the complex nature of implementation pathways, this approach may limit the understanding of the long-term outcomes. Finally, the described effects may not align with the described time horizon of 5 years. AI development speed predictions are highly volatile. Some projections may materialize sooner, while others may never materialize.

## Conclusions

Our analysis systematically explores the multifaceted effects of introducing AI into wellness platforms. The projected futures are not uniform and positive; there are parallel trajectories as well as competing ones. Empowerment may arrive together with risk amplification. Better data governance and user-centric design are needed to protect user autonomy. AI wellness apps are aimed at changing individual behavior, but they operate in a broader social and technological context. Complementary future (and futures) studies should include diverse stakeholders and quantify the trade-offs of these platforms.

## Supplementary material

10.2196/96285 Multimedia Appendix 1 Evolution of first-order consequences.

## Abbreviations

FW: Futures Wheel
OECD: Organisation for Economic Co-operation and Development
