# Supplementary material for: Anticipating the Impact of AI on Diet and Exercise Apps: Foresight Study Applying the Futures Wheel
Source: J Med Internet Res. 2026 Jul 16;28:e96285. doi: 10.2196/96285 (PMC13375074; doi:10.2196/96285)
Supplement: Multimedia Appendix 1 [file jmir-v28-e96285-s001.docx]

**Table S1.** Evolution of first-order consequences.

| Initial expert-generated concepts (subset mapped to first-order effects) * | Final first-order effect |
| --- | --- |
| Hyper-personalized behaviour-change loops  Personalized diet and exercise plans  Dynamic diet-exercise co-optimisation | Personalization of nutrition and fitness plans |
| Wearables and IoT devices | Integration with smart technology |
| Data commodification and privacy contestation  Increased surveillance of eating and movement patterns | Increased privacy and surveillance concerns |
| Employer or insurer access to behavioral data | Data-driven risk profiling and moral hazard |
| Digital-health equity fault lines  New health disparities | Acceleration of health inequalities driven by the digital divide |
| Re-engineered clinical workflows | Incorporation into organizational processes |
| Constant AI coaching and feedback loops | 24/7 health coaching |

* During consolidation, some initially generated concepts were reclassified as second-order effects based on their dependency on primary drivers.

## **Approach**

In this study, we used a qualitative foresight methodology—the FW—to explore the potential medium- to long-term effects of AI integration in consumer wellness applications, specifically diet and exercise apps. The FW is a structured, participatory technique designed to identify and organize the direct and indirect consequences of a defined a central trend by mapping successive layers of impacts. It is an established participatory foresight method where a central trend is defined, and participants create a list of their perceived first- and higher-order consequences expected to emerge from that trend.

We focused the analysis on end-users of the applications rather than other actors such as developers, regulators, and the overall healthcare system. First-order consequences of the trend stem from the central phenomenon and are linked to it with spokes; together they are visualized as a wheel. Second-order consequences were defined as downstream effects arising from each first-order consequence and were represented as a subsequent ring. We explore potential effects within a five-year horizon.

The FW was developed through structured consequence mapping and iterative synthesis conducted by the study’s authors (X.T., G.S., S.L.) as panel members. First-order consequences were generated independently and subsequently discussed in group sessions, during which overlapping or redundant impacts were merged or discarded. All members reached consensus on which consequences to include and how to classify them. The subsequent selection of second-order consequences iteratively followed the same procedure. We redefined the wheel over several iterations to improve its coherence and internal consistency.

To connect the wheel with the existing knowledge base, we expanded the consequences with the prevailing scientific literature, policy reports, and publicly disclosed information of product development efforts. This step helped us contextualize the impacts, without attempting a comprehensive review of all available empirical evidence. The final FW served as the analytical framework for organizing and interpreting the main findings.

**Panel Composition**

The FW was developed by a three-member expert panel consisting of the study’s authors. The panel was intentionally interdisciplinary to capture technical, behavioral, and socio-institutional dimensions of AI integration into wellness applications. The panel included: (1) a biologist with expertise in metabolic health and digital biomarkers, (2) a behavioral economist with expertise in decision science, incentives, and health behavior change and (3) a communication scientist specializing in digital media ecosystems and user–technology interaction. The panel size (n=3) aligns with the exploratory nature of the FW method, which is designed for structured consequence mapping. Members were selected to provide complementary expertise across biological, behavioral, and technical dimensions of AI-enabled wellness systems. To mitigate potential bias, the implemented safeguards included independent generation of first-order consequences, iterative group deliberation, and unanimous inclusion criteria. All panel members had prior research experience related to digital health technologies. Given the exploratory and methodological focus of the study, these three authors themselves served as the elicitation panel.

**Elicitation Procedure**

The FW employed a structured, multi-stage elicitation process conducted over three iterative working sessions (each approximately 60–90 minutes) across a six-week period.

Stage 1: Central Trend Definition
The panel first defined and operationalized the central trend: integration of AI into consumer wellness applications for personalized nutrition and fitness guidance. The scope was limited to consumer-facing digital platforms (e.g., mobile apps integrated with wearables) but excluded clinical decision support systems, and AI integrated in the clinical workflow (e.g., diagnostics).

Stage 2: Independent First-Order Consequence Mapping
Panel members independently generated potential first-order consequences, defined as direct and immediate outcomes resulting from widespread AI-driven personalization. Independent generation was used to reduce anchoring effects and groupthink. Ideas were submitted in writing prior to group discussion. During October–November 2025, panel members used generative AI tools (GPT-5 and Gemini 2.5 Pro) for individual brainstorming. No generative AI was used in manuscript writing beyond the brainstorming stage. AI-generated ideas were critically evaluated, reformulated, and either discarded or validated by the respective member before submitting it to the rest of the panel. These tools were used to widen divergent thinking. Inclusion required consensus independent of AI origin. AI was not used in classification, validation, or final structuring.

Stage 3: Group Deliberation and Expansion
During structured workshops, independently generated consequences were pooled, clarified, and discussed. Duplicates were merged, ambiguities resolved, and conceptual overlaps identified. For each retained first-order consequence, the panel then generated second-order consequences—defined as indirect, systemic, or emergent effects resulting from the first-order impacts.

The group followed a forward-expansion logic: each consequence was examined using the prompt, “If this becomes widespread and normalized, what structural or behavioral changes follow?” We continued this process until a point of theoretical saturation, where no substantially new ideas emerged. Disagreements were resolved through structured discussion. Items that lacked a clear causal justification were either reformulated or removed. No voting system was used; inclusion required unanimous agreement that was reached for all items appearing in the manuscript.

**Consolidation and Thematic Structuring**

Following expansion, second-order consequences were consolidated through thematic coding. Items were grouped into broader domains (e.g., technology, user behavior, infrastructure, and legal/ethical framework). Consolidation decisions required consensus among all three panel members. Disagreements were resolved through discussion. If consensus could not be reached immediately, the item was provisionally retained and revisited in a subsequent session. Final inclusion required unanimous agreement.

To enhance coherence and prevent redundancy, consequences were refined to ensure: i) conceptual distinctiveness, ii) logical traceability from the central trend, and a iii) non-circular causal structure. This consolidation process resulted in a structured two-tier FW comprising first- and second-order consequences.

The evolution from initial expert-generated concepts to consolidated first-order effects is summarized in Supplementary Table S1; some concepts were reclassified as higher-order effects during this process.

## **Analysis**

Following the construction of the FW, an evaluative analysis phase was conducted. Collectively, the participants reviewed all consequences and assessed them for internal consistency and relevance. In this phase, consequences were iteratively revised, allowing impacts to be merged, reframed, reclassified across orders, or removed when deemed redundant or insufficiently linked to the central trend. At a higher level, the analysis also aimed to identify recurring patterns, reinforcing dynamics, and trade-offs across different branches of the wheel. We identified patterns by comparing clusters of consequences, rather than applying any quantitative weighting or prioritization.

Wheel editing followed Peter Wagschal rule of unanimity, whereby impacts are included by consensus. This rule ensures that the wheel’s effects are reasonable, and that conclusions are not too speculative and of limited predicting value. Each effect was analyzed and supported with related references from scientific journals, grey literature and informal sources that capture emerging signals or weak trends. The finalized FW and the identified patterns informed the organization and narrative structure of the upcoming Results section. First-order consequences are presented as primary categories, with associated second-order consequences discussed within each category to illustrate downstream processes.

## **Contextualization**

Supporting literature is used to contextualize and assess the plausibility of these mapped effects.

1. **Personalization of Nutrition and Fitness Plans**

One of the most immediate consequences of AI integration in wellness apps is the increasing degree of personalization in diet and exercise recommendations. Personalization is feasible due to the convergence of complementary technologies. Wearables devices and digital platforms generate a continuous stream of data on users’ physical activity, behavior, and biomarkers, while mobile interfaces facilitate the delivery of feedback and interventions designed to maintain engagement and foster behavior change.

AI systems transform personalized nutrition and fitness by analyzing individual preferences, identifying nutritional needs, and tailoring health objectives accordingly. They leverage machine learning algorithms to generate dietary suggestions and exercise routines tailored to the availability of resources, culture, and individual characteristics. These adapt over time based on user behavior and progress. Through real-time feedback and dynamic optimization, these platforms have been associated with improvements in motivation, adherence, and outcomes in user samples [^[[1]](#endnote-1)^,^[[2]](#endnote-2)^,^[[3]](#endnote-3)^,^[[4]](#endnote-4)^,^[[5]](#endnote-5)^].

Focusing on the impacts on user outcomes, the secondary consequences distinguish the key dimensions of personalization along potentials, limitation and challenges, and spillover effects at the individual level.

- 1. **Improved Health Outcomes**

Emerging evidence suggests that AI-enabled wellness apps can produce significant improvements in physical activity, metabolic markers, and weight regulation. A large-scale platform deployed AI-driven nudges via a graph neural network across 84,764 individuals over 12 weeks. Results of the study included a 6.17% increase in daily steps and a 7.61% increase in weekly moderate-to-vigorous physical activity, with positive engagement metrics [^[[6]](#endnote-6)^]. Another large-scale retrospective cohort study of 944 users demonstrated that an AI-supported mobile app significantly improved key biometric indicators within 33 days. The time in range, for glucose went from 74.7% to 85.5% in healthy users, the average weight reduction was 1.5 kg; and there was a trend toward reduced blood glucose variability [^[[7]](#endnote-7)^]. These studies suggest that AI-enhanced wellness tools may produce health outcomes previously associated with clinical or coach-led interventions, indicating their potential role in scalable, personalized health promotion. They also provide an implementation pathway for harnessing health behavior change theory by identifying the individual users’ behavioral profiles and creating tailored nudges.

Beyond clinical outcomes, behavioral outcomes are evidenced too. A real-world randomized controlled trial utilized a WeChat mini program to evaluate meals pre- and post-consumption through AI-driven nutritional analysis. Results showed a significant reduction in meat intake and an increase in plant-based foods at lunch [^[[8]](#endnote-8)^]. These findings suggest that AI-enabled dietary feedback can shift users' food choices toward more health-conscious eating patterns. At scale, these trends could contribute to public health goals and environmental sustainability.

- 1. **Algorithmic Biases and Challenges**

A growing concern lies in the propensity of algorithmic systems to reinforce discriminatory practices. Algorithmic bias can provoke harms in employment, housing, and healthcare. A study examined biases in consumer mobile health (mHealth) tools—including fitness and diet apps—and found that algorithmic systems may amplify discrimination against marginalized groups if left unchecked [^[[9]](#endnote-9)^]. A separate study identified racial bias in a widely used healthcare algorithm. Black patients classified at the same risk level as white patients were, in fact, more clinically ill [^[[10]](#endnote-10)^]. Left unaddressed, algorithmic bias in intelligent mHealth could evolve into a future state where marginalized populations systematically receive lower-quality wellness support, reinforcing existing health disparities [^[[11]](#endnote-11)^].

- 1. **Mental Health Costs**

The precision of the data generated with AI may lead to mental health strain. A systematic review found that the use of diet and fitness apps may be linked to disorderly eating, body image concerns, and compulsive exercise [^[[12]](#endnote-12)^]. In one of the reviewed studies, approximately half of the participants reported negative experiences related to app use, such as obsessive calorie-counting, dietary restriction, guilt, and other low mood [^[[13]](#endnote-13)^]. AI feedback can promote health-related behaviors but may also trigger an obsession for validation.

Users may prioritize validation from the app over intrinsic motivation or personal well-being. A descriptive survey of 200 users explored how real-time AI feedback loops (e.g., notifications, badges, streaks) can foster technostress, anxiety, addictive behaviors, and loss of autonomy [^[[14]](#endnote-14)^]. Conversely, wellness AI app users might become frustrated with constant monitoring, optimizing, and performance checking. Initial improvements could plateau or reverse due to app burnout, distrust in AI recommendations, or loss of intrinsic motivation [^[[15]](#endnote-15)^].

- 1. **Impact on Food and Exercise Culture**

As AI systems increasingly shape how individuals eat and exercise, they may contribute to gradual cultural shifts. Personalized nutrition and fitness technologies could alter communal practices, potentially eroding social cohesion and transforming long-standing food and exercise traditions. A qualitative report from a food-tech industry workshop highlights industry concerns that AI recipe-generation systems could promote over-standardization and homogenization of meals, overshadowing “happy accidents” that spark novel, culturally diverse dishes [^[[16]](#endnote-16)^]. Excessive dependence on AI-driven personalized nutrition may undermine traditional food cultures and reduce dietary diversity. In a future where individuals are encouraged to follow algorithm-generated meal plans that prioritize nutritional efficiency above all else, diets could become increasingly uniform, centered around a limited set of AI-defined optimal food choices, potentially at the expense of the cultural and sensory aspects of eating [^[[17]](#endnote-17)^].

Personalized nutrition challenges one-diet-fits-all dietary approaches [^[[18]](#endnote-18)^]. Thus, at the individual level, a readjustment of social dynamics is expected. Individuals may eat different foods and at different times from those around them, disrupting communal meal experiences that promote social cohesion. Divergent personalized exercise habits can also redefine social ties. For instance, instead of participating in shared group activities like team sports or community fitness classes, individuals might follow AI-prescribed routines tailored to their biometrics and preferences, often alone, at specific times, or even in virtual environments. Certain traditional sports or physical activities reliant on group participation may face decline.

1. **24/7 Health Coaching**

Digital health interventions are available around the clock. AI-driven virtual assistants can feasibly deliver lifestyle changes with measurable improvements in physical activity, diet, sleep, and body composition [^[[19]](#endnote-19)^]. Advancing evidence-based digital health coaching will depend on standardizing how engagement and coaching delivery are measured and contextualized [^[[20]](#endnote-20)^].

Coaching, by default, is a two-sided market. Digitalization will impact the relationship between users and service providers by replacing the latter. Thus, the second-order consequences must cover how these changes impact the two sides. In particular, users will face a different choice set in terms of accessibility, barriers to adaptation, and agency, while conventional providers will face a technological disruption, the extent of which remains to be seen.

- 1. **Greater Accessibility to Expert-Level Guidance**

The demand for support in improving nutrition, fitness, and overall health far exceeds the supply of qualified coaches and healthcare professionals. AI is helping bridge this gap by providing expert-level guidance that is more accessible, scalable, and efficient than traditional approaches [^[[21]](#endnote-21)^]. Future research should focus on enhancing real-time adaptability, integrating physiological monitoring, and strengthening AI-human collaboration to maximize the precision and effectiveness of AI-driven exercise recommendations [^[[22]](#endnote-22)^].

- 1. **Increased Health Literacy**

AI can support real-time health education that adapts to varying literacy levels [^[[23]](#endnote-23)^]. Chatbots and virtual assistants improve users’ understanding of health information and encourage preventive behaviors [^[[24]](#endnote-24)^]. Fitness and diet apps make information more understandable, interactive, and culturally accessible. Such apps help users connect their daily choices with tangible health outcomes, strengthening both knowledge and behavior change.

- 1. **Reduced Human Agency**

AI-based tools are taking on tasks traditionally performed by dietitians and trainers. In a recent study, ChatGPT showed the ability to classify foods for renal nutrition planning, and performed well on basic tasks, but lacked the nuanced, individualized recommendations offered by dietitians [^[[25]](#endnote-25)^]. In any case, conversational agents can be valuable complements to human coaching. A different study shows that AI can support user self-management, help in setting personally meaningful goals, and offer encouragement. Some users even compared the experience favorably with interactions with human coaches. In short, AI can shoulder certain aspects of health and wellness coaching, easing the demand on human professionals [^[[26]](#endnote-26)^].

High reliance on AI introduces risks such as reduced decision making and erosion of expertise and responsibility. In health contexts, this could translate into a less refined analysis of consequences [^[[27]](#endnote-27)^]. Technological and regulatory safeguards and a tight collaboration between developers and practitioners can help avoid non-desired outcomes [^[[28]](#endnote-28)^]. For AI-powered diet and exercise apps users, automatic following of AI guidance could lead to a non-autonomous lifestyle.

- 1. **De-platforming vs Augmentation of Health and Fitness Professionals**

The proliferation of AI-enabled platforms may adversely impact wellness professionals. Dietitians may find less opportunities to use their skills among the sheer deluge of AI-based food classifiers, meal planners or educational apps. The same is expected to happen to fitness coaches when competing with exercise prescription and motivational apps. They are recommended to adapt their skillset to stay relevant [^[[29]](#endnote-29)^].

In parallel, AI may augment the arsenal of practitioners to help their patients and clients [^[[30]](#endnote-30)^]. AI can potentially enhance the specificity with which practitioners understand intra-individual variability, including interaction effects between nutrients, temporal changes in response to nutrients, and context-dependent efficacy across physiological states [^[[31]](#endnote-31)^]. AI-assisted analysis may help understand nutrients’ impact on circadian-impacted biomarkers such as cortisol or insulin over time. Behavioral insights, dynamic intervention adjustment, early risk detection, user phenotyping, and longitudinal personalization are some of the tools available to practitioners [^[[32]](#endnote-32)^,^[[33]](#endnote-33)^,^[[34]](#endnote-34)^,^[[35]](#endnote-35)^]. Longevity and preventive health interventions, which require long-term commitment, may benefit from a digital n-of-1 approach. As subjects evolve over time, optimal interventions should be modulated dynamically [^[[36]](#endnote-36)^,^[[37]](#endnote-37)^,^[[38]](#endnote-38)^,^[[39]](#endnote-39)^]. Together, AI, apps and wearables can track sleep, heart rate variability, glucose and ketone levels, among others. In addition, longitudinal monitoring of biomarker dynamics may serve as a motivational signal that sustains engagement in long interventions [^[[40]](#endnote-40)^,^[[41]](#endnote-41)^]. Rather than a replacement, AI may work as an extension of the practitioner’s toolkit, allowing a more individualized care.

From a user perspective, these two potential outcomes may have similar implications. The de-platforming of professionals could reduce access to human oversight. AI augmentation of professionals may result in more adaptive interventions. Both trends may coexist to varying degrees, giving rise to a heterogeneous wellness landscape in which wearable-driven solutions operate alongside personal coaching positioned as an augmented, premium-priced service [^[[42]](#endnote-42)^].

1. **Integration with Smart Technology**

Various wearable sensors—such as smart textiles, oral devices, lens-based monitors, microfluidic skin patches, and pressure sensors—already enable high-accuracy personalized monitoring of physiological and chemical parameters. These technologies support continuous assessment of health status and support timely, data-driven interventions for prevention, management, and treatment [^[[43]](#endnote-43)^].

At a broader scale, the combined potential of the Internet of Things (IoT) and AI allows smart systems can collect health data, analyze it, integrate it with other data streams (agriculture, supply chain, industry, retail), and produce decisions that improve the standard of living [^[[44]](#endnote-44)^]. AI-powered supply chain platforms already drive predictive analytics, smart cart systems, inventory management, and shopper behavior modelling, shaping product availability and customer choice [^[[45]](#endnote-45)^].

Within the wellness domain, the integration of AI with smart technologies—wearables, sensors, continuous glucose monitors (CGMs)—is expected to enhance the effectiveness of nutrition and fitness recommendations. IoT devices transform real-time health data into relevant metrics of physical activity, while AI-driven recommendation systems can combine data from multiple sensors to generate adaptive individualized dietary [^[[46]](#endnote-46)^]. As algorithms gain access to currently untapped data sources, subsequent health improvements are expected [^[[47]](#endnote-47)^].

Integration occurs between existing and in-development technologies. Thus, the relevant second-order consequences cover the key hardware and software solutions that may form the key components of this ecosystem.

- 1. **Smart Glasses: On-Demand Nutritional Content Analysis**

As a second-order effect of AI integration, emerging vision-based systems are transforming dietary assessment, a domain long constrained by self-reporting bias and participant burden. Researchers employed low-cost wearable cameras to develop EgoDiet, building an egocentric vision-based pipeline to automatically estimate portion sizes. The platform demonstrated superior accuracy compared to professional dietitians, achieving lower error rates in portion size estimation [^[[48]](#endnote-48)^]. Another platform, DietGlance employs eyeglasses to detect ingestion episodes through multimodal inputs. The AI identifies food items and their quantity, analyzes the meal’s nutritional value and recommends diet changes [^[[49]](#endnote-49)^].

A similar platform has nearly 80% accuracy in food recognition [^[[50]](#endnote-50)^]. LLMs like ChatGPT 4o and Gemini 1.5 Pro have already reached user self-assessment precision [^[[51]](#endnote-51)^]. Today, these passive and frictionless systems have shown higher accuracy than human self-reporting. They are expected to improve and their adoption may expand in the mid-term.

- 1. **Smart Wearables: Enhanced Data Tracking and Analysis**

Smart wearables are gaining importance in AI-wellness systems when guiding users’ behavior and monitoring their activity. Objective measurements of step count, heart rate, or estimations of energy expenditure, evidence this system’s superior accuracy and reliability over self-reporting [^[[52]](#endnote-52)^]. Wearables’ capabilities to promote health are discussed in a recent meta-analysis, concluding that physical intervention mediated by these devices may prevent obesity [^[[53]](#endnote-53)^]. By reducing hospital visits and enabling remote monitoring, smart wearables can lower healthcare costs [^[[54]](#endnote-54)^].

- 1. **Smart Apps: Gamification and AI-Driven Adaptive Challenges**

Keeping the user engaged with a wellness app in the long-term is necessary to achieve a sustained effect. Gamification has arisen as a way to increase the user’s interaction with the app and improve its effectiveness [^[[55]](#endnote-55)^]. Rewards, challenges and goal tracking have been shown to increase physical activity [^[[56]](#endnote-56)^,^[[57]](#endnote-57)^]. These elements work better as individual tests rather than with a one-size-fits-all approach [^[[58]](#endnote-58)^], which makes AI-powered gamification a key contributor to an intervention’s feasibility [^[[59]](#endnote-59)^]. AI-driven adaptive game design holds promise for serious games [^[[60]](#endnote-60)^].

- 1. **Smart Ambiances: Home, Health and Daily Routines**

AI presence is reaching every environment and context, linking home, health and habits for continuous wellness. By integrating a vision-based object detection system along with supply chain and user food interest prediction systems, complete automation of groceries ordering can be achieved [^[[61]](#endnote-61)^]. Visual reasoning models are already capable of engaging in natural conversations with users about refrigerator contents and their properties (e.g., freshness) [^[[62]](#endnote-62)^]. Another platform leveraging large language models (LLMs) has demonstrated the ability to generate tailored weekly meal plans that incorporate constraints such as USDA dietary guidelines and ingredient availability [^[[63]](#endnote-63)^]. We argue that such AI systems could recommend healthy meals based on available ingredients at home and user preferences, or suggest alternative purchases to enhance nutritional value or overall user experience.

Environmental sensors coupled with a location and status estimation algorithm can determine house occupants’ activity status and their location [^[[64]](#endnote-64)^]. Another intervention design features IoT devices coupled to a mobile app to modify sedentary behavior [^[[65]](#endnote-65)^]. Prediction of falls and management of chronic diseases becomes possible with the advent of AI, where AI-driven algorithms make inferences on future states based on past and current behavioral patterns recorded by sensors embedded in physical spaces [^[[66]](#endnote-66)^].

1. **Increased Privacy and Surveillance Concerns**

As a first-order effect of AI-integration in wellness technologies, escalating privacy and surveillance risks have become a persistent concern. As far back as 2015, Huckvale et al. found that a vast majority (89%) of evaluated mHealth apps transmitted user data to external services without proper encryption, exposing sensitive information to potential breaches [^[[67]](#endnote-67)^]. A 2022 study captured growing concerns around the data use of mHealth technologies such as smart sensors and apps, particularly among older adults cautious of opaque data practices [^[[68]](#endnote-68)^]. In the same year, an empirical evaluation of leading mental health apps uncovered critical privacy vulnerabilities such as unnecessary permissions, insecure data handling, and pervasive third-party data sharing, increasing risks of user profiling and identifiability [^[[69]](#endnote-69)^].

As the study of privacy and surveillance reveals a complex relationship between stakeholders, we approach it by identifying these stakeholders, the underlying mechanisms, and possible pathways to resolve legitimate user concerns. The secondary consequences uncover these intricate pathways between stakeholders, emphasizing the immediate challenges and the possible solutions.

- 1. **Privacy Erosion in Everyday Activities**

Digitalization is a double-edged sword: convenience, efficiency and speed are invariably linked to an inherent risk of privacy loss. A study analyzing 796 Android health apps found that nearly 25% didn’t implement proper privacy policies, and most apps collecting sensitive data did not comply with GDPR regulations [^[[70]](#endnote-70)^]. A different study found that mental health apps stored and transmitted data insecurely, while allowing third-party profiling [64].

- 1. **Data Monetization by Tech Companies**

Users are rightly concerned about data exploitation by wellness apps. Tech companies have a great incentive in accessing personal data. There are concerns that data collection and monetization constitute key components of business models of major technology companies offering fitness tools [^[[71]](#endnote-71)^]. A 2025 study found that 80% of the dominant fitness apps (e.g. Strava, Fitbit, Nike Training Club) share health information with data brokers and advertisers [^[[72]](#endnote-72)^].

- 1. **Robust Hardware and Software Safeguards**

Growing privacy and security concerns accompany the expanding use of ML into a wide range of application areas. Data owners increasingly use cloud computing to perform training and inference on sensitive information, but vulnerabilities to data breaches and compromised computational integrity persist. Privacy-preserving algorithms are needed, with robust hardware-based security mechanisms. There is ongoing research on data obfuscation strategies that provide provable data privacy and computation integrity in the cloud servers [^[[73]](#endnote-73)^]. Additionally, a novel platform leverages hardware (e.g., electrocardiogram wearables) noise as a dual-purpose resource to enhance ML robustness and secure data [^[[74]](#endnote-74)^].

- 1. **Policy-Driven Alternatives to Corporate Data Models**

Rising awareness of privacy risks is stimulating the emergence of public alternatives to corporate data ecosystems. A 2024 Organisation for Economic Co-operation and Development (OECD) report supports the implementation of the Privacy Guidelines alongside the AI Principles. By advocating for international co-operation, the report aims to guide the development of AI systems that respect and support privacy. The OECD.AI Expert Group on AI, Data, and Privacy promotes working on sector-specific issues at the intersection of AI and privacy, such as in health, employment, or finance [^[[75]](#endnote-75)^].

- 1. **Data Cooperatives and Personal Vaults**

User engagement presents an alternative pathway to resolve privacy concerns. Hafen et al. advocate for the creation of democratically governed, non-profit personal data cooperatives as a model for managing data sharing and donation. This approach empowers individuals to retain control over their own data and to share its economic value, particularly in the context of health research and precision medicine. Proponents argue that users may become the primary aggregators and beneficiaries of their data, taking power away from corporate legal entities [^[[76]](#endnote-76)^]. Although some progress has been made in shifting corporate governance practices, a broader movement is needed to enable communities to collectively shape and oversee the AI systems that affect their daily lives [^[[77]](#endnote-77)^].

1. **Data-Driven Risk Profiling and Moral Hazard**

Companies justify the collection of data from employees and customers by adducing its need for workplace wellness program optimization or for tailoring insurance offers based on individual health behaviors. The whole picture is more concerning, as risk profiling categorizes individuals by virtue of algorithms that may not consider social, economic or medical constraints. This creates a moral hazard where trying to influence behavior may punish the less apt and reinforce disparities [^[[78]](#endnote-78)^]. The problem is aggravated by firms sharing data with third parties, disregarding fairness, transparency, and consent [^[[79]](#endnote-79)^]. Without deliberate design constraints embedded at the point of product development and procurement, these dynamics are likely to be replicated — and amplified — as AI wellness tools scale across health systems and insurance markets.

- 1. **Lifestyle-Based Underwriting**

Adaptive underwriting, which uses biometric and lifestyle data as inputs, is being progressively favored by health insurance companies. Real-time activity levels and diet patterns inform insurers about risks incurred by customers and how to incentivize them to modify their behavior. A 2025 survey found that the insured consider unfair the practices where insurers can influence the premium fairness [^[[80]](#endnote-80)^]. A key implementation challenge lies in explainability: users subject to AI-informed underwriting decisions should, in principle, be able to understand which behavioral inputs triggered a change in their premium, yet most current platforms do not offer this level of transparency to end users.

The new status quo in the insurance industry, where AI use may become prevalent, will require new guidelines for insurance companies. The National Association of Insurance Commissioners (NAIC) instructions on AI transparency, human oversight and risk management are being adopted by U.S. states [^[[81]](#endnote-81)^]. Several of them are passing laws that forbid the exclusive use of AI to deny coverage, determine benefits or set incentives [^[[82]](#endnote-82)^].

- 1. **Corporate Wellness Optimization Using Employee Data**

Corporations and employees’ interests converge in regards to workforce health, at least on the surface. Companies can make use of AI to foster health, which improves productivity and reduces healthcare costs [^[[83]](#endnote-83)^]. Apps like Louvity use predictive analytics to identify work-related stressors, and then provide tailored wellness plans in real time. Future plans include integrating VR and IoT [^[[84]](#endnote-84)^]. The usual health data privacy concerns are present among employees, whose resistance needs to be addressed if these solutions are to become mainstream [56,57].

1. **Incorporation into Organizational Processes**

Wellness AI is progressively being integrated into public and private organizations. Health, education and data science institutions connected through public-private partnerships are developing AI apps for chronic disease prevention [^[[85]](#endnote-85)^]. Hospitals and universities collaborate on wellness AI programs that leverage lifestyle data to modify clinical workflows and optimize care [^[[86]](#endnote-86)^]. Wellness and insurance companies provide personalized interventions to employees [^[[87]](#endnote-87)^]. For users, this will translate into an increase in AI exposure, which will shape users’ interactions with institutions.

- 1. **Re-Engineered Clinical Workflows**

AI and technology-enabled clinical workflow redesigns are being embedded into routine healthcare delivery, especially in virtual care. Institutions such as Houston Methodist and Emory presented real-world examples of AI-driven workflow transformation that improves care coordination and efficacy [^[[88]](#endnote-88)^]. Medicine–engineering collaborations are advancing precision care for chronic disease management through tools for real-time monitoring and data-driven interventions [^[[89]](#endnote-89)^]. AI-driven alert systems can support real-time triage with high diagnostic accuracy [^[[90]](#endnote-90)^]. AI-based clinical alerts decrease alert burden and increase identification of unsuitable prescriptions [^[[91]](#endnote-91)^]. Users will experience continuous health monitoring, faster triage in emergency situations, and clinical recommendations.

- 1. **Lifestyle Medicine Transformation**

AI has the potential to shift lifestyle medicine from reactive interventions to proactive care. Chang & Liu reviewed how AI-enabled precision interventions in diet, physical activity, and chronic disease prevention can help individuals sustain healthier behaviors and improve long-term outcomes [^[[92]](#endnote-92)^]. Saeed & Nashwan envision digital twin simulations enabling individuals to test lifestyle changes before adopting them [^[[93]](#endnote-93)^].

- 1. **Increased Regulatory Scrutiny and Classification Debates**

FDA's initial classification of many AI wellness tools as "general wellness products", exempt from stringent regulatory oversight, has come under scrutiny as these apps begin to influence health decisions [^[[94]](#endnote-94)^]. The lack of clear regulatory standards creates uncertainty among users about the reliability, safety, and clinical validity of these tools.

This is concerning when wellness apps are used in medical capacity. Certain AI chatbots have been found to respond in ways that increase psychological distress [^[[95]](#endnote-95)^]. These gaps highlight the need for clearer governance frameworks to ensure that wellness apps are evaluated according to their real impact on users [^[[96]](#endnote-96)^,^[[97]](#endnote-97)^].

1. **Acceleration of Health Inequality Driven by the Digital Divide**

Smart devices remain unaffordable for a considerable portion of the population. The producers pricing strategy may involve a lump-sum price, a monthly subscription fee, or a combination of both. Subscription fees are made possible by the necessity to gain access to online platforms. However, their combination constitutes two-part pricing which enables price discrimination and the extraction of consumer surplus [^[[98]](#endnote-98)^]. These economic constraints limit global access to digital interventions [^[[99]](#endnote-99)^]. However, free distribution of digital tools [^[[100]](#endnote-100)^] will not solve the issue unless broader structural reforms happen, such as programs to increase digital literacy [^[[101]](#endnote-101)^].

The main barrier to adoption in this area is financial and well-understood. However, the solutions require substantial efforts and will remain unresolved in the near future. Thus, the second-order consequences entail plausible and likely solution pathways toward a more inclusive digital ecosystem.

- 1. **Open-Source Tools**

Free alternatives to proprietary software exist for many widely used applications, and are now starting to develop for AI tools. Institutions, developers and researchers’ collaboration accelerates the development of AI tools that are cost-effective [^[[102]](#endnote-102)^]. Several examples exist. OpenHealth is a personal health AI-assistant that saves diet and activity data locally [^[[103]](#endnote-103)^]. MONAI (Medical Open Network for AI) is an open-source app for medical imaging [^[[104]](#endnote-104)^]. Hugging Face Transformers is an open-source library with pre-trained models for ML tasks that can be used for clinical text mining or medical coding automation [^[[105]](#endnote-105)^].

- 1. **Protection and Inclusion of Marginalized Populations**

Regulations are evolving to address the protection needs of marginalized users of AI tools. National laws can follow guidance from the World Health Organization’s 2021 document on AI for health governance, which identifies six core ethical principles: transparency, inclusiveness, accountability, equity, autonomy, and safety [^[[106]](#endnote-106)^]. In the U.S., the Department of Health and Human Services mandated entities using AI-driven decision support tools to assess bias, impact and possible discriminatory outcomes [^[[107]](#endnote-107)^].

- 1. **User-Centric Design**

App design must put the user at the center. This is true for any kind of user, but specially needed for people with disabilities, who should ideally be involved from the development stage [^[[108]](#endnote-108)^]. Lived experience insights are invaluable for optimal usability and reduced algorithmic bias. A publication from the United Nations Regional Information Centre stresses the importance of an intersectional approach to tackle issues arising from overlapping marginalities [^[[109]](#endnote-109)^].

- 1. **Subsidized AI-Supported Programs**

Subsidizing AI programs and devices is a straightforward measure to diminish the digital divide. The Institute for Global Change recommends governments to set a target of 30% of the population in health programs involving wearables by 2027 [^[[110]](#endnote-110)^]. Free wearables are well received by underserved populations when accompanied by behavioral and economic incentives [^[[111]](#endnote-111)^].

- 1. **Community Digital Literacy Initiatives**

Free smart devices might be necessary, but not sufficient. Marginalized collectives tend to have low digital and health literacy. Digital tools with positively impact these communities’ health outcomes if enough trust is gained to implement literacy programs [^[[112]](#endnote-112)^]. Only with their involvement in design and implementation will the community understand and use these resources. Similarly, rural populations require their own, targeted measures to close the urban-rural gap in digital health access [^[[113]](#endnote-113)^].

References

1. Gabarron E, Larbi D, Rivera-Romero O, Denecke K. Human factors in AI-driven digital solutions for increasing physical activity: scoping review. *JMIR Hum Factors*. 2024;11:e55964. doi:10.2196/55964. [↑](#endnote-ref-1)
2. An R, Shen J, Wang J, Yang Y. A scoping review of methodologies for applying artificial intelligence to physical activity interventions. *J Sport Health Sci*. 2024;13(3):428-441. doi:10.1016/j.jshs.2023.09.010. [↑](#endnote-ref-2)
3. Agrawal K, Goktas P, Kumar N, Leung MF. Artificial intelligence in personalized nutrition and food manufacturing: a comprehensive review of methods, applications, and future directions. *Front Nutr.* 2025;12. doi:10.3389/fnut.2025.1636980. [↑](#endnote-ref-3)
4. Roars Inc. The Role of AI in Personalized Nutrition and Fitness Plans. Our Journal. Published July 16, 2024. Accessed August 18, 2025. https://www.roarsinc.com/our-journal/the-role-of-ai-in-personalized-nutrition-and-fitness-plans/. [↑](#endnote-ref-4)
5. Irwin S. AI in Fitness: The Future of Personalized Workouts and Nutrition Plans. The Fitness Zone. Published July 26, 2024. Accessed August 18, 2025. https://fitness.edu.au/the-fitness-zone/ai-in-fitness-the-future-of-personalized-workouts-and-nutrition-plans/. [↑](#endnote-ref-5)
6. Chiam J, Lim A, Nott C, Mark N, Teredesai A, Shinde S. Co-pilot for health: personalized algorithmic AI nudging to improve health outcomes. arXiv. Preprint posted online January 19, 2024. Available from: https://arxiv.org/abs/2401.10816. [↑](#endnote-ref-6)
7. Veluvali A, Dehghani Zahedani A, Hosseinian A, et al. Impact of digital health interventions on glycemic control and weight management. *NPJ Digit Med*. 2025;8:20. doi:10.1038/s41746-025-01050-2. [↑](#endnote-ref-7)
8. Liu H, Feng J, Shi Z, et al. Effects of a novel applet-based personalized dietary intervention on dietary intakes: a randomized controlled trial in a real-world scenario. *Nutrients*. 2024;16(4):565. doi:10.3390/nu16040565. [↑](#endnote-ref-8)
9. Gloria, K., Rastogi, N., & DeGroff, S. (2022). Bias Impact Analysis of AI in Consumer Mobile Health Technologies: Legal, Technical, and Policy. arXiv preprint arXiv:2209.05440. [↑](#endnote-ref-9)
10. Obermeyer Z, Powers B, Vogeli C, Mullainathan S. Dissecting racial bias in an algorithm used to manage the health of populations. *Science* 366, 447-453 (2019). DOI:10.1126/science.aax2342. [↑](#endnote-ref-10)
11. Commission on Social Determinants of Health. *Closing the Gap in a Generation: Health Equity Through Action on the Social Determinants of Health.* World Health Organization; 2008. [↑](#endnote-ref-11)
12. Anderberg I, Kemps E, Prichard I. The link between the use of diet and fitness monitoring apps, body image and disordered eating symptomology: A systematic review. *Body Image*. 2025;52:101836. doi:10.1016/j.bodyim.2024.101836. [↑](#endnote-ref-12)
13. Honary M, Bell B, Clinch S, Wild S, McNaney R. Understanding the role of healthy eating and fitness mobile apps in the formation of maladaptive eating and exercise behaviors in young people. *JMIR Mhealth Uhealth*. 2019;7(6):e14239. doi:10.2196/14239. [↑](#endnote-ref-13)
14. Adanyin A. AI‑Driven Feedback Loops in Digital Technologies: Psychological Impacts on User Behaviour and Well‑Being. arXiv Preprint. 2024 Oct 30. Available from: arXiv:2411.09706. [↑](#endnote-ref-14)
15. Dhir A, Nijjer S, Luo X (Robert), Kaur P. Wearable discontinuance: Pathways in the volitional information systems discontinuance process. *Information & Management*. 2024;61(8):104038. doi:10.1016/j.im.2024.104038. [↑](#endnote-ref-15)
16. Nosowitz D. I attended a workshop on the impact of AI on the food world—here’s what we discussed. The Spoon. Published May 30, 2024. Accessed August 18, 2025. https://thespoon.tech/i-attended-a-workshop-on-the-impact-of-ai-on-the-food-world-heres-what-we-discussed/ [↑](#endnote-ref-16)
17. Ethical implications of AI in personalized nutrition. PRISM. Accessed August 18, 2025. https://prism.sustainability-directory.com/scenario/ethical-implications-of-ai-in-personalized-nutrition/ [↑](#endnote-ref-17)
18. Roman S, Campos-Medina L, Leal-Mercado L. Personalized nutrition: the end of the one-diet-fits-all era. *Front Nutr.* 2024;11. doi:10.3389/fnut.2024.1370595. Accessed December 1, 2025. https://www.frontiersin.org/journals/nutrition/articles/10.3389/fnut.2024.1370595. [↑](#endnote-ref-18)
19. Maher CA, Davis CR, Curtis RG, Short CE, Murphy KJ. A Physical Activity and Diet Program Delivered by Artificially Intelligent Virtual Health Coach: Proof-of-Concept Study. *JMIR Mhealth Uhealth*. 2020;8(7):e17558. Published 2020 Jul 10. doi:10.2196/17558. [↑](#endnote-ref-19)
20. Loughnane C, Laiti J, O'Donovan R, Dunne PJ. Systematic review exploring human, AI, and hybrid health coaching in digital health interventions: trends, engagement, and lifestyle outcomes*. Front Digit Health.* 2025;7:1536416. Published 2025 Apr 24. doi:10.3389/fdgth.2025.1536416. [↑](#endnote-ref-20)
21. Lady World Press. Enhancing health and wellness with AI. The Lady of the World. May 2, 2025. Accessed September 30, 2025. [https://theladyoftheworld.com/2025/05/02/enhancing-health-and-wellness-with-ai/](https://theladyoftheworld.com/2025/05/02/enhancing-health-and-wellness-with-ai/?utm_source=chatgpt.com). [↑](#endnote-ref-21)
22. Puce L, Bragazzi NL, Currà A, Trompetto C. Harnessing Generative Artificial Intelligence for Exercise and Training Prescription: Applications and Implications in Sports and Physical Activity—A Systematic Literature Review. *Applied Sciences*. 2025; 15(7):3497. https://doi.org/10.3390/app15073497. [↑](#endnote-ref-22)
23. Meskó B, Görög M. A short guide for medical professionals in the era of artificial intelligence. *NPJ Digit Med.* 2020;3:126. Published 2020 Sep 24. doi:10.1038/s41746-020-00333-z [↑](#endnote-ref-23)
24. Sultan AA, Goktas BB. The role of artificial intelligence in increasing the health literacy of patients. Published online 2024. Accessed June 25, 2025. https://www.researchgate.net/publication/381852088_The_Role_of_Artificial_Intelligence_In_Increasing_the_Health_Literacy_of_Patients. [↑](#endnote-ref-24)
25. Güner E, Ülker MT. Can artificial intelligence replace dietitians? A conversation with ChatGPT. J *Food Nutr Gastronomy*. 2024;3(1):49–56. doi:10.58625/jfng-2474. [↑](#endnote-ref-25)
26. Mitchell EG, Desai P, Smaldone A, et al. T2 Coach: a qualitative study of an automated health coach for diabetes self-management. In: Proceedings of the 2025 CHI Conference on Human Factors in Computing Systems (CHI '25). New York, NY: Association for Computing Machinery; 2025:Article 357. doi:10.1145/3706598.3714404. [↑](#endnote-ref-26)
27. Ahmad SF, Han H, Alam MM, et al. Impact of artificial intelligence on human loss in decision making, laziness and safety in education. *Humanit Soc Sci Commun*. 2023;10:311. doi:10.1057/s41599-023-01787-8. [↑](#endnote-ref-27)
28. Abdelwanis M, Alarafati H, Tammam M, Simsekler MCE. Exploring the risks of automation bias in healthcare artificial intelligence applications: a Bowtie analysis. *J Saf Sci Resil*. 2024;5. doi:10.1016/j.jnlssr.2024.06.001. [↑](#endnote-ref-28)
29. Glebova E, Madsen D, Mihaľová P, Géczi G, Mittelman A, Jorgič B. Artificial intelligence development and dissemination impact on the sports industry labor market. *Frontiers in Sports and Active Living*, Volume 6, 2024. [↑](#endnote-ref-29)
30. Topol EJ. High-performance medicine: the convergence of human and artificial intelligence. *Nat Med*. 2019;25(1):44-56. doi:10.1038/s41591-018-0300-7. [↑](#endnote-ref-30)
31. Zeevi D, Korem T, Zmora N, et al. Personalized Nutrition by Prediction of Glycemic Responses. Cell. 2015;163(5):1079-1094. doi:10.1016/j.cell.2015.11.001. [↑](#endnote-ref-31)
32. Dunn J, Kidzinski L, Runge R, et al. Wearable sensors enable personalized predictions of clinical laboratory measurements. *Nat Med*. 2021;27(6):1105-1112. doi:10.1038/s41591-021-01339-0. [↑](#endnote-ref-32)
33. Alzghaibi H. Adoption barriers and facilitators of wearable health devices with AI integration: a patient-centred perspective. *Front Med (Lausanne)*. 2025;12:1557054. Published 2025 Apr 3. doi:10.3389/fmed.2025.1557054. [↑](#endnote-ref-33)
34. Uppuluri V. Real-time monitoring of patient adherence using artificial intelligence. Int J Health Sci. 2025;8(3):52-68. doi:10.47941/ijhs.3120. [↑](#endnote-ref-34)
35. Berry SE, Valdes AM, Drew DA, et al. Human postprandial responses to food and potential for precision nutrition. *Nat Med*. 2020;26(6):964-973. doi:10.1038/s41591-020-0934-0. [↑](#endnote-ref-35)
36. Pantuck AJ, Lee DK, Kee T, Wang P, Lakhotia S, Silverman MH, et al. Modulating BET bromodomain inhibitor ZEN-3694 and enzalutamide combination dosing in a metastatic prostate cancer patient using CURATE.AI, an artificial intelligence platform. *Adv Ther*. 2018;1(6):1800104. [↑](#endnote-ref-36)
37. Kee T, Weiyan C, Blasiak A, Wang P, Chong JK, Chen J, et al. Harnessing CURATE.AI as a digital therapeutics platform by identifying N-of-1 learning trajectory profiles. Adv Ther. 2019;2(9):1900023. [↑](#endnote-ref-37)
38. Chong LM, Wang P, Lee VV, Vijayakumar S, Tan HQ, Wang FQ, et al. Radiation therapy with phenotypic medicine: towards N-of-1 personalization. Br J Cancer. 2024;131(1):1-10. [↑](#endnote-ref-38)
39. Blasiak A, Truong AT, Foo N, Tan LW, Kumar KS, Tan S-B, et al. Personalized dose selection

    platform for patients with solid tumors in the PRECISE CURATE. AI feasibility trial. *npj Precision*

    *Oncology*. 2025;9(1):49. [↑](#endnote-ref-39)
40. Wang P, Tadeo X, Chew HSJ, et al. N-of-1 health optimization: Digital monitoring of biomarker dynamics to gamify adherence to metabolic switching. *PNAS Nexus*. 2024;3(6):pgae214. Published 2024 May 30. doi:10.1093/pnasnexus/pgae214. [↑](#endnote-ref-40)
41. Wang P, Foo N, Su C, et al. DELTA: fortifying human biological resilience with an N=1 digital health and dynamic biomarker protocol. medRxiv. Published online February 10, 2026. doi:10.64898/2026.02.10.26345969. [↑](#endnote-ref-41)
42. Tirole J. The theory of industrial organization. MIT Press; 1988. [↑](#endnote-ref-42)
43. Shajari S, Kuruvinashetti K, Komeili A, Sundararaj U. The Emergence of AI-Based Wearable Sensors for Digital Health Technology: A Review. Sensors. 2023; 23(23):9498. https://doi.org/10.3390/s23239498. [↑](#endnote-ref-43)
44. Alahi MEE, Sukkuea A, Tina FW, et al. Integration of IoT-Enabled Technologies and Artificial Intelligence (AI) for Smart City Scenario: Recent Advancements and Future Trends. *Sensors (Basel).* 2023;23(11):5206. Published 2023 May 30. doi:10.3390/s23115206. [↑](#endnote-ref-44)
45. Bhide M. Smarter carts: how AI is transforming grocery shopping. News-Medical. Published February 7, 2024. Accessed August 18, 2025. https://www.news-medical.net/health/Smarter-carts-How-AI-is-transforming-grocery-shopping.aspx. [↑](#endnote-ref-45)
46. Tsolakidis D, Gymnopoulos LP, Dimitropoulos K. Artificial Intelligence and Machine Learning Technologies for Personalized Nutrition: A Review. Informatics. 2024; 11(3):62. https://doi.org/10.3390/informatics11030062. [↑](#endnote-ref-46)
47. Brankovic A, Hendrie GA. Perspectives, challenges and future of artificial intelligence in personalised nutrition research. *Proceedings of the Nutrition Society*. Published online 2025:1-9. doi:10.1017/S0029665125100657. [↑](#endnote-ref-47)
48. Lo FPW, Qiu J, Jobarteh ML, et al. AI-enabled wearable cameras for assisting dietary assessment in African populations. *NPJ Digit Med*. 2024;7:356. doi:10.1038/s41746-024-01346-8. [↑](#endnote-ref-48)
49. Jiang Z, Zhao R, Lin L, et al. DietGlance: dietary monitoring and personalized analysis at a glance with knowledge empowered AI assistant. arXiv. Preprint posted online February 2, 2025. doi:10.48550/arXiv.2502.01317. [↑](#endnote-ref-49)
50. Han M, Chen J, Zhou Z. NutrifyAI: an AI-powered system for real-time food detection, nutritional analysis, and personalized meal recommendations. arXiv. Preprint posted online August 20, 2024. doi:10.48550/arXiv.2408.10532. [↑](#endnote-ref-50)
51. Fridolfsson J, Sjöberg E, Thiwång M, Pettersson S. Performance evaluation of 3 large language models for nutritional content estimation from food images. *Curr Dev Nutr*. 2025;9(10):107556. doi:10.1016/j.cdnut.2025.107556. [↑](#endnote-ref-51)
52. Natalucci V, Marmondi F, Biraghi M, Bonato M. The effectiveness of wearable devices in non-communicable diseases to manage physical activity and nutrition: where we are? *Nutrients*. 2023;15(4):913. doi:10.3390/nu15040913. [↑](#endnote-ref-52)
53. Wang W, Cheng J, Song W, Shen Y. The effectiveness of wearable devices as physical activity interventions for preventing and treating obesity in children and adolescents: systematic review and meta-analysis. *JMIR Mhealth Uhealth*. 2022;10(4):e32435. doi:10.2196/32435. [↑](#endnote-ref-53)
54. Steinhubl SR, Muse ED, Topol EJ. The emerging field of mobile health. *Sci Transl Med*. 2015;7(283):283rv3. doi:10.1126/scitranslmed.aaa3487. [↑](#endnote-ref-54)
55. Rehman U, Abbasi AZ, Ting DH, Hassan M, Khair N. Exploring the impact of gamified experiences on user engagement in fitness apps: a GAMEFULQUEST perspective. *IEEE Trans Eng Manag*. 2024;71:3613-3628. doi:10.1109/TEM.2023.3347231. [↑](#endnote-ref-55)
56. Xu L, Shi H, Shen M, et al. The effects of mHealth-based gamification interventions on participation in physical activity: systematic review. *JMIR Mhealth Uhealth*. 2022;10(2):e27794. doi:10.2196/27794. [↑](#endnote-ref-56)
57. Yang Y, Hu H, Koenigstorfer J. Effects of gamified smartphone applications on physical activity: a systematic review and meta-analysis. *Am J Prev Med*. 2022;62(4):602-613. doi:10.1016/j.amepre.2021.10.015. [↑](#endnote-ref-57)
58. Cappelen AW, Charness G, Ekström M, Gneezy U, Tungodden B. Exercise improves academic performance. *J Polit Econ*. [Published online ahead of print]. doi:10.1086/738251. [↑](#endnote-ref-58)
59. Gao Y, Zhang J, He Z, Zhou Z. Feasibility and usability of an artificial intelligence-powered gamification intervention for enhancing physical activity among college students: quasi-experimental study. *JMIR Serious Games*. 2025;13:e65498. doi:10.2196/65498. [↑](#endnote-ref-59)
60. Tolks D, Schmidt JJ, Kuhn S. The Role of AI in Serious Games and Gamification for Health: Scoping Review. *JMIR Serious Games*. 2024;12:e48258. Published 2024 Jan 15. doi:10.2196/48258. [↑](#endnote-ref-60)
61. Mereddy D. Vision-based automatic groceries tracking system – smart homes. arXiv. Preprint posted online December 19, 2023. doi:10.48550/arXiv.2312.12486. [↑](#endnote-ref-61)
62. Gudovskiy D, Han G, Yamaguchi T, Tsukizawa S. Smart home appliances: chat with your fridge. arXiv. Preprint posted online December 20, 2019. doi:10.48550/arXiv.1912.09589. [↑](#endnote-ref-62)
63. Khamesian S, Arefeen A, Carpenter SM, Ghasemzadeh H. NutriGen: personalized meal plan generator leveraging large language models to enhance dietary and nutritional adherence. arXiv. Preprint posted online February 29, 2025. doi:10.48550/arXiv.2502.20601. [↑](#endnote-ref-63)
64. Wang Y, Yalcin A, VandeWeerd C. Health and wellness monitoring using ambient sensor networks. *Journal of Ambient Intelligence and Smart Environments*. 2020;12(2):139-151. doi:10.3233/AIS-200553. [↑](#endnote-ref-64)
65. Huang Y, Benford S, Price D, Patel R, Li B, Ivanov A, Blake H. Using Internet of Things to Reduce Office Workers’ Sedentary Behavior: Intervention Development Applying the Behavior Change Wheel and Human-Centered Design Approach. *JMIR Mhealth Uhealth* 2020; 8(7):e17914. doi: [10.2196/17914.](https://doi.org/10.2196/17914)  [↑](#endnote-ref-65)
66. Haque, A., Milstein, A. & Fei-Fei, L. Illuminating the dark spaces of healthcare with ambient intelligence. *Nature* 585, 193–202 (2020). https://doi.org/10.1038/s41586-020-2669-y. [↑](#endnote-ref-66)
67. Huckvale K, Prieto JT, Tilney M, Benghozi PJ, Car J. Unaddressed privacy risks in accredited health and wellness apps: a cross-sectional systematic assessment. *BMC Med*. 2015;13:214. Published 2015 Sep 7. doi:10.1186/s12916-015-0444-y. [↑](#endnote-ref-67)
68. Schroeder T, Haug M, Gewald H. Data Privacy Concerns Using mHealth Apps and Smart Speakers: Comparative Interview Study Among Mature Adults. *JMIR Form Res*. 2022;6(6):e28025. Published 2022 Jun 13. doi:10.2196/28025. [↑](#endnote-ref-68)
69. Iwaya LH, Babar MA, Rashid A, Wijayarathna C. On the privacy of mental health apps: An empirical investigation and its implications for app development. *Empir Softw Eng*. 2023;28(1):2. doi:10.1007/s10664-022-10236-0. Epub 2022 Nov 8. PMID: 36407814; PMCID: PMC9643945. [↑](#endnote-ref-69)
70. Fan M, Yu L, Chen S, et al. An empirical evaluation of GDPR compliance violations in Android mHealth apps. arXiv 2020 Aug 13; Available from: arXiv:2008.05864. [↑](#endnote-ref-70)
71. Nosthoff AV, Maschewski F, Couldry N. Big Tech is exploiting the mental health crisis to monetize your data. Jacobin. Published December 1, 2023. Accessed June 23, 2025. https://jacobin.com/2023/12/big-tech-mental-health-data-collection-colonization. [↑](#endnote-ref-71)
72. Surfshark VPN. 80% of top fitness apps share user data with third parties. Surfshark. Published January 7, 2025. Accessed June 23, 2025. https://surfshark.com/research/chart/fitness-apps-privacy?srsltid=AfmBOorny9SnWI0BY1R3F_2dgS2NooRXku5GunV0nEB19YnAhM6K7t52. [↑](#endnote-ref-72)
73. Hashemi H, Wang Y, Annavaram M. DarKnight: an accelerated framework for privacy and integrity preserving deep learning using trusted hardware. arXiv. 2022 Jun 30. doi:10.48550/arXiv.2207.00083 [↑](#endnote-ref-73)
74. Chamon C, Sarkar A, Abbott AL. Noise Driven AI Sensors: secure healthcare monitoring with PUFs. arXiv. 2025 Jun 5. doi:10.48550/arXiv.2506.05135. [↑](#endnote-ref-74)
75. Organisation for Economic Co-operation and Development (OECD). *AI, Data Governance and Privacy.* OECD Publishing; 2024 Jun. Available from: https://www.oecd.org/content/dam/oecd/en/publications/reports/2024/06/ai-data-governance-and-privacy_2ac13a42/2476b1a4-en.pdf. [↑](#endnote-ref-75)
76. Hafen E. Personal Data Cooperatives – A New Data Governance Framework for Data Donations and Precision Health. 2019 Jan 16. In: Krutzinna J, Floridi L, editors. *The Ethics of Medical Data Donation [Internet].* Cham (CH): Springer; 2019. Chapter 9. PMID: 32091856. [↑](#endnote-ref-76)
77. Hubbard S. Cooperative Paradigms for Artificial Intelligence. Ash Center for Democratic Governance and Innovation, Harvard Kennedy School; 2024. Accessed June 25, 2025. https://ash.harvard.edu/resources/cooperative-paradigms-for-artificial-intelligence [↑](#endnote-ref-77)
78. OECD. *Digital tools for health and wellness in insurance [Policy Paper].* Paris: OECD Publishing; 2024 Mar 13. doi:10.1787/d3764184-en. [↑](#endnote-ref-78)
79. IS Partners LLC. Data Privacy at Risk with Health and Wellness Apps. IS Partners LLC; 2023. Accessed August 29, 2025. https://www.ispartnersllc.com/blog/data-privacy-health-apps/. [↑](#endnote-ref-79)
80. Press G. Preventative AI is poised to transform health insurance. Forbes. Published June 24, 2025. Accessed June 25, 2025. https://www.forbes.com/sites/gilpress/2025/06/24/preventative-ai-is-poised-to-transform-health-insurance. [↑](#endnote-ref-80)
81. National Association of Insurance Commissioners. Artificial intelligence. NAIC. Published December 2023. Accessed June 25, 2025. https://content.naic.org/insurance-topics/artificial-intelligence. [↑](#endnote-ref-81)
82. Morgan Lewis. Ain’t done yet: states continue to craft rules to manage AI tools in healthcare. Morgan Lewis Insights. Published April 22, 2025. Accessed June 25, 2025. https://www.morganlewis.com/pubs/2025/04/aint-done-yet-states-continue-to-craft-rules-to-manage-ai-tools-in-healthcare. [↑](#endnote-ref-82)
83. Korrapati R. AI-driven personalized employee wellness programs: enhancing engagement and outcomes. Published online 2025. Accessed June 25, 2025. https://www.researchgate.net/publication/388926986_AI-Driven_Personalized_Employee_Wellness_Programs_Enhancing_Engagement_and_Outcomes [↑](#endnote-ref-83)
84. Omose O, Ikuyinminu KS. Enhancing employee wellness and mitigating corporate burnout through predictive analytics: a case study of Louvity’s innovative application design and implementation. *Iconic Res Eng J*. 2024;8(6):32. ISSN: 2456-8880. [↑](#endnote-ref-84)
85. AIM-AHEAD Program. Public-private partnerships to improve population health using AI/ML. AIM-AHEAD. Published 2024. Accessed June 25, 2025. https://www.aim-ahead.net/programs/public-private-partnerships-to-improve-population-health-using-ai-ml-program. [↑](#endnote-ref-85)
86. Rice University. Rice, Houston Methodist launch pathbreaking Digital Health Institute to transform future of care. Rice News. Published January 22, 2024. Accessed June 25, 2025. https://news.rice.edu/news/2024/rice-houston-methodist-launch-pathbreaking-digital-health-institute-transform-future. [↑](#endnote-ref-86)
87. Financial Times. Wellness meets insurance: Howden adopts CloudFit for employee health. Financial Times. Published April 12, 2024. Accessed June 25, 2025. https://www.ft.com/content/d7c1b4b8-2ff6-4b05-8721-1f22aa9527e3. [↑](#endnote-ref-87)
88. Schwamm LH, Pletcher S, Erskine A. AI and Technology Enabled Clinical Workflow Redesign. Telemed Rep. 2024;5(1):415-420. Published 2024 Dec 23. doi:10.1089/tmr.2024.0079. [↑](#endnote-ref-88)
89. Dong C, Ji Y, Fu Z, et al. Precision management in chronic disease: an AI empowered perspective on medicine-engineering crossover*. iScience*. 2025;28(3):112044. doi:10.1016/j.isci.2025.112044. [↑](#endnote-ref-89)
90. Tahernejad A, Sahebi A, Salehi Sahl Abadi A, et al. Application of artificial intelligence in triage in emergencies and disasters: a systematic review. *BMC Public Health*. 2024;24:3203. doi:10.1186/s12889-024-20447-3. [↑](#endnote-ref-90)
91. Graafsma J, Murphy RM, van de Garde EMW, et al. The use of artificial intelligence to optimize medication alerts generated by clinical decision support systems: a scoping review. *J Am Med Inform Assoc*. 2024;31(6):1411-1422. doi:10.1093/jamia/ocae076. [↑](#endnote-ref-91)
92. Chan KHK, Liu S. The role of artificial intelligence in advancing lifestyle medicine research. In: Mechanick JI, Kushner RF, eds. *Lifestyle Medicine*. Cham: Springer; 2025. doi:10.1007/978-3-031-82888-1_17. [↑](#endnote-ref-92)
93. Saeed D K, Nashwan A J (June 08, 2025) Harnessing Artificial Intelligence in Lifestyle Medicine: Opportunities, Challenges, and Future Directions. *Cureus* 17(6): e85580. doi:10.7759/cureus.85580. [↑](#endnote-ref-93)
94. Abulibdeh R, Celi LA, Sejdić E. The illusion of safety: A report to the FDA on AI healthcare product approvals. *PLOS Digit Health*. 2025 Jun 5;4(6):e0000866. doi: 10.1371/journal.pdig.0000866. PMID: 40471897; PMCID: PMC12140231. [↑](#endnote-ref-94)
95. Yoon SC, An JH, Choi JS, Chang JH, Jang YJ, Jeon HJ. Digital Psychiatry with Chatbot: Recent Advances and Limitations. *Clin Psychopharmacol Neurosci*. 2025;23(4):542-550. doi:10.9758/cpn.25.1346. [↑](#endnote-ref-95)
96. De Freitas J, Cohen IG. The health risks of generative AI-based wellness apps. *Nat Med*. 2024;30(5):1269-1275. doi:10.1038/s41591-024-02943-6. [↑](#endnote-ref-96)
97. Meskó B, Topol EJ. The imperative for regulatory oversight of large language models (or generative AI) in healthcare. *NPJ Digit Med*. 2023;6(1):120. Published 2023 Jul 6. doi:10.1038/s41746-023-00873-0. [↑](#endnote-ref-97)
98. Pindyck RS, Rubinfeld DL. *Microeconomics*. Global ed. Pearson; 2018. [↑](#endnote-ref-98)
99. Fabbrizio A, Fucarino A, Cantoia M, De Giorgio A, Garrido ND, Iuliano E, Reis VM, Sausa M, Vilaça-Alves J, Zimatore G, Baldari C, Macaluso F. Smart Devices for Health and Wellness Applied to Tele-Exercise: An Overview of New Trends and Technologies Such as IoT and AI. *Healthcare (Basel)*. 2023 Jun 20;11(12):1805. doi: 10.3390/healthcare11121805. PMID: 37372922; PMCID: PMC10298072. [↑](#endnote-ref-99)
100. Savage M. Call for poor and disabled to be given NHS fitness trackers. The Guardian. Published May 4, 2019. Accessed August 29, 2025. https://www.theguardian.com/inequality/2019/may/04/fitbits-nhs-reduce-inequality-health-disability-poverty. [↑](#endnote-ref-100)
101. American Heart Association. Equitable access to digital technologies may help improve cardiovascular health. Newsroom. Published April 7, 2025. Accessed August 29, 2025. https://newsroom.heart.org/news/equitable-access-to-digital-technologies-may-help-improve-cardiovascular-health. [↑](#endnote-ref-101)
102. Cake.ai. Open-source healthcare AI tools. Published 2024. Accessed August 29, 2025. https://www.cake.ai/blog/open-source-healthcare-ai-tools. [↑](#endnote-ref-102)
103. OpenHealthForAll. OpenHealth: AI‑enabled personal health assistant. GitHub. Updated 2024. Accessed June 25, 2025. https://github.com/OpenHealthForAll/open-health. [↑](#endnote-ref-103)
104. MONAI Project. Medical Open Network for AI (MONAI). Accessed August 29, 2025. https://monai.io/. [↑](#endnote-ref-104)
105. Hugging Face. Transformers documentation. Accessed August 29, 2025. https://huggingface.co/docs/transformers/en/index. [↑](#endnote-ref-105)
106. World Health Organization. Ethics and Governance of Artificial Intelligence for Health: WHO Guidance. World Health Organization; 2021. Accessed June 25, 2025. https://www.who.int/publications/i/item/9789240029200. [↑](#endnote-ref-106)
107. Bipartisan Policy Center. Navigating AI in health care: HHS’s nondiscrimination final rule is in effect. Bipartisan Policy Center. Published July 5, 2024. Accessed June 25, 2025. https://bipartisanpolicy.org/blog/navigating-ai-in-health-care-hhss-nondiscrimination-final-rule-is-in-effect. [↑](#endnote-ref-107)
108. El Morr C, Singh D, Sawhney V, Fernandes S, El-Lahib Y, Gorman R. Exploring the intersection of AI and inclusive design for people with disabilities. *Stud Health Technol Inform*. 2024;316:556-559. doi:10.3233/SHTI240475. PMID: 39176803. [↑](#endnote-ref-108)
109. United Nations Regional Information Centre. Building an accessible future for all: AI and the inclusion of persons with disabilities. UNRIC. Published October 13, 2023. Accessed June 25, 2025. https://unric.org/en/building-an-accessible-future-for-all-ai-and-the-inclusion-of-persons-with-disabilities. [↑](#endnote-ref-109)
110. Institute for Global Change. The watch is ticking: a five-year plan to harness wearable health tech. Published November 21, 2022. Accessed March 17, 2026. https://institute.global/insights/public-services/watch-ticking-five-year-plan-harness-wearable-health-tech. [↑](#endnote-ref-110)
111. Soliño-Fernandez D, Ding A, Bayro-Kaiser E, et al. Willingness to adopt wearable devices with behavioral and economic incentives by health insurance wellness programs: results of a US cross-sectional survey with multiple consumer health vignettes. *BMC Public Health*. 2019;19:1649. doi:10.1186/s12889-019-7920-9. [↑](#endnote-ref-111)
112. Sørensen K. Fostering digital health literacy to enhance trust and improve health outcomes. *Comput Methods Programs Biomed Update*. 2024;5:100140. doi:10.1016/j.cmpbup.2024.100140 [↑](#endnote-ref-112)
113. Ji H, Dong J, Pan W, et al. Associations between digital literacy, health literacy, and digital health behaviors among rural residents: evidence from Zhejiang, China. *Int J Equity Health*. 2024;23:68. doi:10.1186/s12939-024-02150-2. [↑](#endnote-ref-113)
